# Synthèse et étude structurale de Na_9_Cr(MoO_4_)_6_

**DOI:** 10.1107/S2056989015005976

**Published:** 2015-04-02

**Authors:** Wassim Dridi, Ines Ennajeh, Mohamed Faouzi Zid

**Affiliations:** a Laboratoire de Matériaux et Cristallochimie, Faculté des Sciences de Tunis, Université de Tunis El Manar, 2092 El Manar Tunis, Tunisia

**Keywords:** crystal structure, molybdate(VI), CrO_6_ octa­hedron, quaternary systems, open framework

## Abstract

The title compound, nona­sodium chromium(III) hexa­kis[molybdate(VI)], Na_9_Cr(MoO_4_)_6_ was prepared by solid-state reactions. The basic structure units are isolated polyhedral clusters composed of a central CrO_6_ octa­hedron sharing vertices with six MoO_4_ tetrahedra to form an open framework in which the Na^+^ cations are bound to the free vertices of the MoO_4_ tetra­hedra. The Cr^3+^ cation has site symmetry of 32 (6*a*), one Na atom sits on a twofold axis (18*e*), and all other atoms are at general positions. The bond-valance-sum model confirms the expected values of ion charges. The title compound is isotypic with Na_9_Sc(MoO_4_)_6_ and Na_9_Fe(MoO_4_)_6_. It is compared and discussed with similar structures.

## Abstract 2

In Na_9_Cr(MoO_4_)_6_ the basic structure units are isolated polyhedral clusters composed of a central CrO6 octa­hedron sharing vertices with six MoO4 to form an open (0D) framework in which the Na^+^ cations are oriented at the free vertices of the MoO4 tetra­hedra.

## Contexte chimique   

Ces dernières années, plusieurs équipes de recherche s’intéressent à l’étude des systèmes quaternaires de type *A*–*M*–Mo–O (*A* = cation monovalent et *M* = métal de transition). Les molybdates présentent plusieurs domaines d’applications: matériaux laser (Khal’baeva *et al.*, 2013 ▸; Hanuza & Maczka, 1994 ▸), ferroélectriques (Isupov, 2005 ▸; Khal’baeva *et al.*, 2012 ▸), piézoélectriques, catalyseurs pour la synthése organique, superionique, liants à haute température, matrices pour la fixation des isotopes à vie longue et autres matériaux (Kotova & Kozhevnikova, 2003 ▸).

Il est nécessaire de noter que les phases rhomboédriques ont un intérêt particulier car elles présentent une cristallochimie similaire avec des composés du type NASICON, qui sont caractérisés par des propriétés superioniques (Kotova & Kozhevnikova, 2003 ▸). C’est dans ce cadre, que nous avons choisi l’exploration des systèmes quaternaires, très peu étudiés à notre connaissance, *A*–Cr–Mo–O (*A* = ion monovalent). Une nouvelle phase de formulation Na_9_Cr(MoO_4_)_6_ a été obtenue par réaction à l’état solide à 798 K.

## Commentaire structurelle   

**Figure 1:**
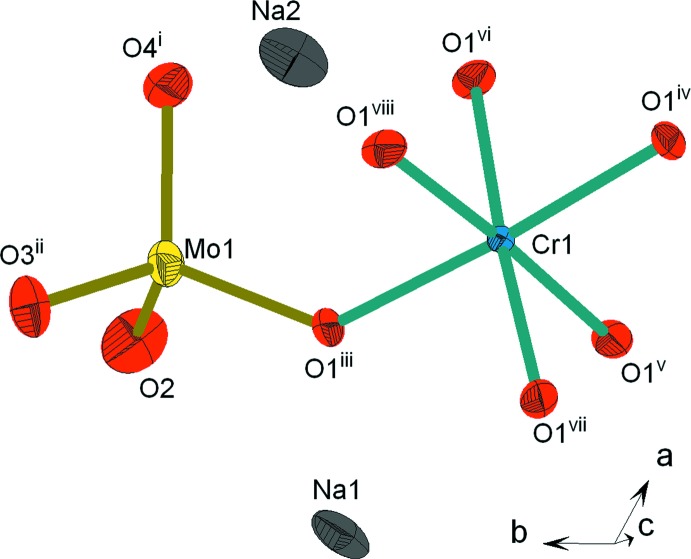
Représentation des polyédres de coordination de l’unité structurale dans Na_9_Cr(MoO_4_)_6_. Les éllipsoïdes ont été définis avec 50% de probabilité. [Codes de symétrie: (i) −*x* + 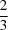, −*x* + *y* + 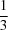, −*z* + 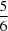; (ii) −*y* + 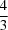, −*x* + 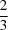, *z* − 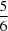; (iii) *y* − 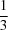, *x* + 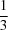, −*z* + 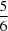; (iv) *y* − 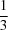, *x* + 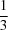, −*z* + 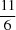; (v) −*y* + 1, *x* − *y* + 1, *z*; (vi) −*x* + *y*, −*x* + 1, *z*; (vii) *x* − *y* + 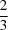, −*y* + 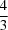, −*z* + 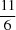; (viii) −*x* + 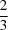, −*x* + *y* + 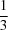, −*z* + 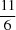.]

**Figure 2:**
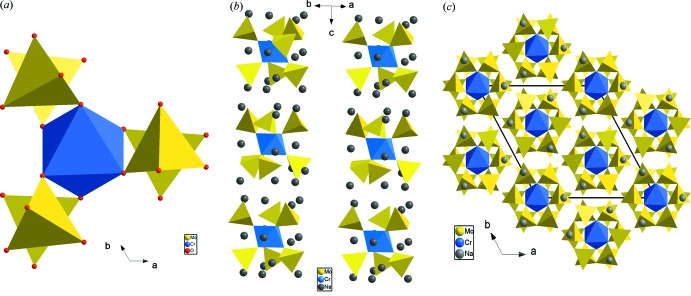
(*a*) Projection d’un cluster [Cr(MoO_4_)_6_]^9−^, (*b*) représentation des unités structurales de Na_9_Cr(MoO_4_)_6_, (*c*) projection de la structure de Na_9_Cr(MoO_4_)_6_ dans le plan (001).

L’unité structurale dans la phase Na_9_Cr(MoO_4_)_6_ est construite à partir d’un octa­èdre CrO_6_ relié par mise en commun d’un sommet à un tétraèdre MoO_4_. La compensation de charges est assurée par les cations Na^+^ (Fig. 1 ▸). Dans la charpente anion­ique chaque octa­èdre CrO_6_ partage ses six sommets avec six tétraèdres MoO_4_ différents pour conduire aux clusters [Cr(MoO_4_)_6_]^9−^ (Fig. 2 ▸ *a*). Ces derniers, dirigés selon [001], prennent une disposition laissant libre des espaces où résident les cations Na^+^ (Fig. 2 ▸ *b*). La répartition de l’ensemble de ces unités selon les trois directions de l’espace engendre une charpente anionique zérodimensionnelle (0D) dans laquelle les cations Na^+^ sont orientés vers les sommets libres des tétraèdres MoO_4_ (Fig. 2 ▸ *c*).

L’examen des facteurs géométriques révèle que les distances Mo—O et Cr—O dans respectivement les tétraèdres MoO_4_ et les octa­èdres CrO_6_ sont similaires à celles rencontrées dans la littérature (Sarapulova *et al.*, 2009 ▸; Tsyrenova *et al.*, 2009 ▸; Gicquel-Mayer *et al.*, 1981 ▸; Bensaid *et al.*, 2013 ▸; Averbuch-Pouchot *et al.*, 1981 ▸). En effet, dans le tétraèdre MoO_4_ les distances Mo—O sont situées dans la gamme 1,723 (4)–1,793 (4) Å. La plus longue liaison Mo—O corres­pond à l’oxygène du pont mixte Cr–O1–Mo. Les distances Cr—O sont toutes égales à 1,966 (3) Å (tableau 1 ▸). Les angles O—Mo—O, situés entre 108,2 (2)—111,8 (2)° correspondent à des tétraèdres MoO_4_ presque réguliers.

De plus, le calcul des sommes de valences de liaison (BVS), utilisant la formule empirique de Brown (2002 ▸) conduit aux valeurs des charges des ions suivants: Mo1 (6,166), Cr1 (3,119), Na1 (1,021), Na2 (0,986) ce qui confirme les degrés d’oxydation des différents ions existant dans la phase étudiée. Un examen bibliographique montre que le matériau obtenu est isostructural aux composés ayant une formulation analogue Na_9_Sc(MoO_4_)_6_ (Savina, Solodovnikov *et al.*, 2013 ▸) et Na_9_Fe(MoO_4_)_6_ (Savina, Morozov *et al.*, 2013 ▸). C’est deux derniers cristallisent dans le même système rhomboédrique mais présentent une symétrie plus réduite groupe *R* 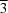. En effet, ils possèdent une unité asymétrique différente de celle de notre composé. Elle est construite à partir de trois atomes de sodium et d’un octa­èdre *M*O_6_ (*M* = Sc, Fe) qui est relié par mise en commun des sommets à deux tétraèdres MoO_4_ différents. La jonction de ces unités par partage de sommets conduit à une charpente similaire à celle du composé obtenu.

## Enquête de base de données   

**Figure 3:**
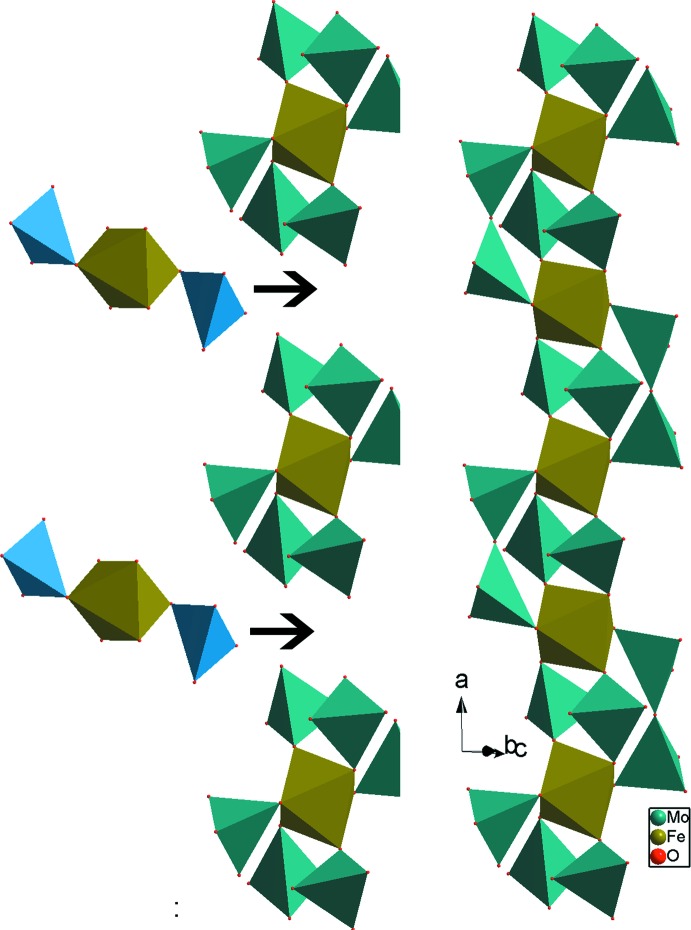
Représentation d’un ruban mettant en évidence la connection entre les unités [Fe(MoO_4_)_6_] de la structure de Rb_3_FeMo_4_O_15_.

**Figure 4:**
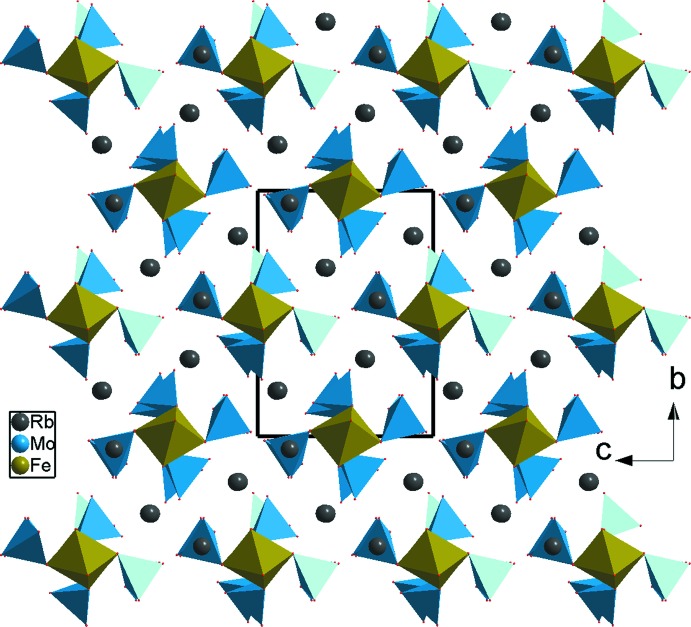
Projection de la structure de Rb_3_FeMo_4_O_15_ selon *a*.

**Figure 5:**
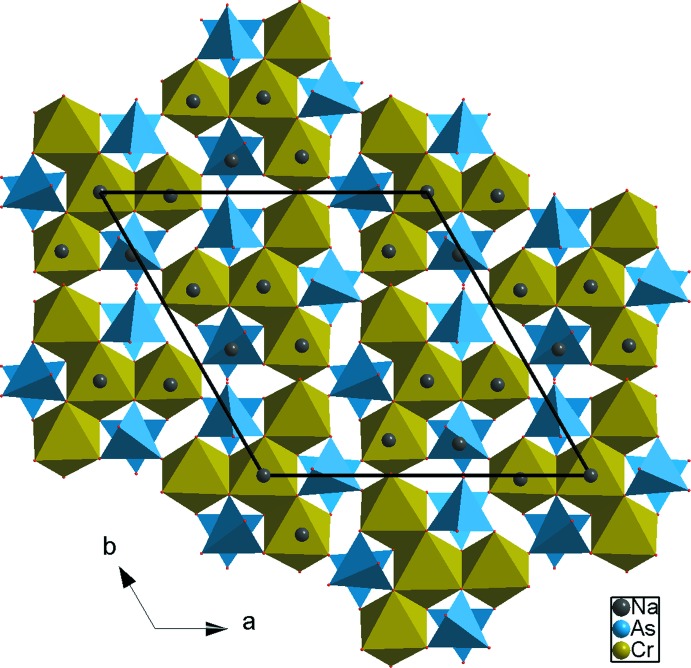
Présentation, selon [001], du réseau tridimensionnel du composé Na_3,5_Cr_1,83_(AsO_4_)_3_.

**Figure 6:**
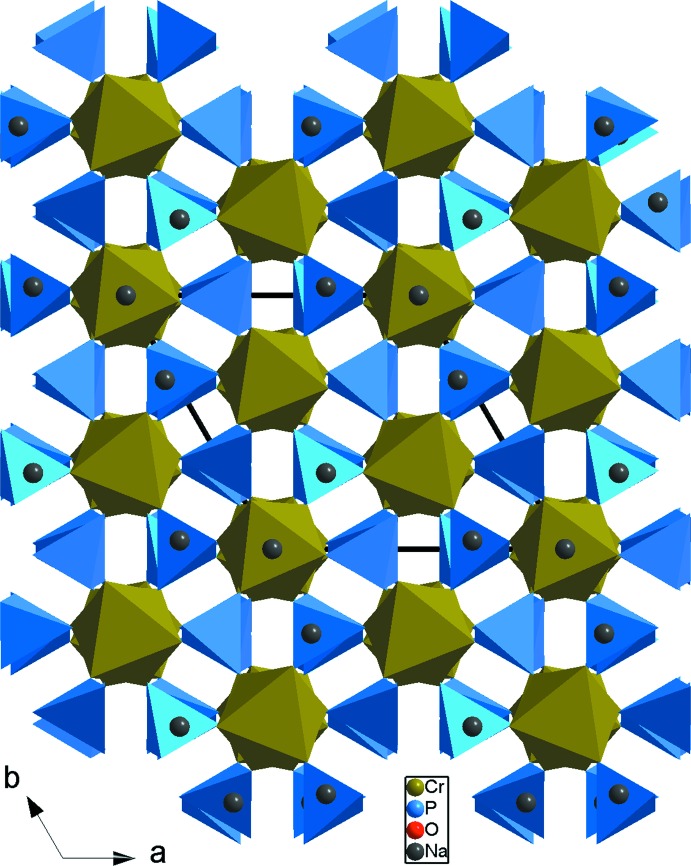
Projection, selon *c*, de la structure du Na_3_Cr_2_(PO_4_)_3_.

La comparaison de la structure de Na_9_Cr(MoO_4_)_6_ avec celles rencontrées dans la littérature montre une certaine analogie avec les composés Rb_3_FeMo_4_O_15_ qui cristallise dans le système monoclinique groupe d’espace *P*2_1_/*c* (Khal’baeva *et al.*, 2010 ▸), Na_3,5_Cr_1,83_(AsO_4_)_3_ (Fakhar Bourguiba *et al.*, 2013 ▸) et Na_3_Cr_2_(PO_4_)_3_ (Genkina *et al.*, 1991 ▸) cristallisant dans le même groupe d’espace *R* 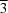 *c*. Le composé Rb_3_FeMo_4_O_15_ est formé par des clusters [Fe(MoO_4_)_6_]^9−^ ayant le même arrangement des polyèdres que celui du cluster [Cr(MoO_4_)_6_]^9−^ existant dans notre structure. De plus, dans Rb_3_Fe(MoO_4_)_2_Mo_2_O_7_, les deux clusters sont liés à l’aide d’une unité linéaire FeMo_2_O_12_ moyennant des ponts mixtes de type Mo–O–Fe et aussi des ponts simples Mo–O–Mo faisant apparaître des groupements dimolybdates Mo_2_O_7_ (Fig. 3 ▸). Il en résulte, des rubans disposés parallèlement à la direction [100] qui présente à leur tour une charpente unidimen­sionnelle possédant des espaces inter-rubans où résident les cations monovalents Rb^+^ (Fig. 4 ▸). Un examen de la charpente anionique du composé Na_3,5_Cr_1,83_(AsO_4_)_3_ révèle la présence des unités formulaires [Cr_4_(AsO_4_)_6_]^6−^. En effet, elles sont formées par des unités [Cr1(AsO_4_)_6_] similaires à celles de notre phase mais dans laquelle chaque octa­èdre central Cr1O_6_ est lié, en plus de partage d’arêtes, à respectivement trois octa­èdres CrO_6_ différents. Ces unités se connectent moyennant des ponts mixtes de type As–O–Cr formant ainsi un réseau tridimensionnel (Fig. 5 ▸). La charpente anionique du composé Na_3_Cr_2_(PO_4_)_3_ présente des unités de type [Cr(PO_4_)_6_]^15−^ possédant une disposition de polyèdres similaire à celle de notre phase. Dans le phosphate Na_3_Cr_2_(PO_4_)_3_, chaque unité se lie par formation de ponts mixtes P–O–Cr avec les unités voisines pour conduire à une charpente tridimensionnelle (Fig. 6 ▸).

## Synthèse et cristallisation   

**Figure 7:**
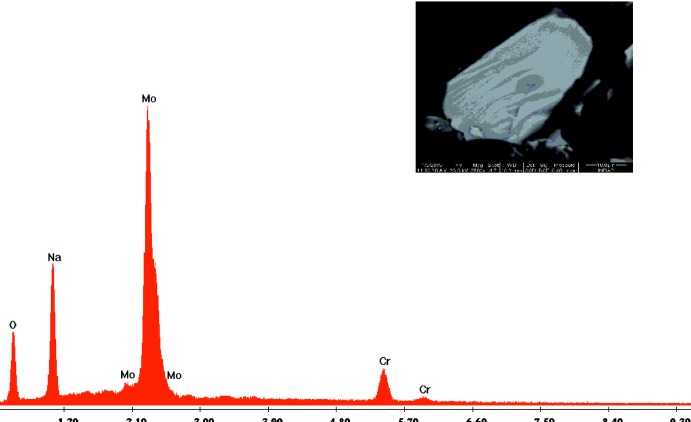
Morphologie et analyse qualitative d’un cristal de Na_9_Cr(MoO_4_)_6_.

Les cristaux relatifs à Na_9_Cr(MoO_4_)_6_ ont été obtenus par réaction à l’état solide à partir des réactifs: Na_2_CO_3_ (PROLABO, 70128, 99,6%), Cr(NO_3_)_3_·9H_2_O (FLUKA, 60832, 99,0%) et (NH_4_)_6_Mo_7_O_24_·4H_2_O (SIGMA-ALDRICH, 13301, 99,0%) pris dans les proportions telques Na:Cr:Mo = 9:0,5:6. Après un broyage effectué dans un mortier en agate, le mélange a été mis dans un creuset en porcelaine, puis préchauffé à l’air à 453 K pendant 24 heures en vue d’éliminer les composés volatils. Il est ensuite porté jusqu’à une température voisine de celle de la fusion à 798 K. Le mélange est abandonné à cette température pendant quelques jours pour favoriser la germination et la croissance des cristaux. Par la suite, il a subi en premier lieu un refroidissement lent (5°/demi journée) jusqu’à 748 K puis rapide (50°/h) jusqu’à la température ambiante. Des cristaux de couleur rouge de taille suffisante pour les mesures des intensités sont obtenus. La morphologie et l’analyse élementaire, ont été faites au moyent d’un microscope électronique à balayage de type FEI *QUANTA* 200. Cette analyse confirme la présence des éléments attendus: Na, Cr, Mo et l’­oxyène (Fig. 7 ▸).

## Affinement   

Les conditions expérimentales de la collecte des données et le résultat final de l’affinement sont rassemblés dans le tableau 2 ▸. En effet, la structure a été résolue et affinée, sans ambiguïté dans le groupe d’espace *R* 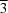 *c* par les méthodes directes utilis­ant la chaíne de programmes *SHELXS97* (Sheldrick, 2008 ▸) inclue dans le système *WinGX* publication (Farrugia, 2012 ▸), partant de la formule Na_3_Cr_1_Mo_3_O_12_. Les premiers cycles d’affinements ont permis de localiser la plupart des atomes. Un examen de la Fourier différence et en se basant sur des considérations géométriques et de neutralité électrique, l’un des atomes d’oxygène a été remplacé par un sodium. L’affinement de tous les paramètres variables conduit à des ellipsoïdes bien définis. Les densités d’électrons maximum et minimum restants dans la Fourier-différence finale sont acceptables et sont situées respectivement à 0,70 Å de O4 et à 1,06 Å de Mo1.

## Supplementary Material

Crystal structure: contains datablock(s) I. DOI: 10.1107/S2056989015005976/ru2063sup1.cif

Structure factors: contains datablock(s) I. DOI: 10.1107/S2056989015005976/ru2063Isup2.hkl

CCDC reference: 1055835

Additional supporting information:  crystallographic information; 3D view; checkCIF report

## Figures and Tables

**Table 1:** Longueurs des liaisons slectionnes ()

Mo1O4^i^	1.723(4)	Na1O4	2.649(4)
Mo1O2	1.730(4)	Na2O2	2.308(4)
Mo1O3^ii^	1.745(4)	Na2O2^iv^	2.384(5)
Mo1O1^iii^	1.793(4)	Na2O4^v^	2.414(5)
Cr1O1	1.966(3)	Na2O3^vi^	2.473(5)
Na1O1	2.364(4)	Na2O3^v^	2.559(4)
Na1O3	2.407(4)	Na2O4^vi^	2.989(5)

Symmetry codes: (i) 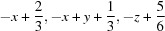; (ii) 
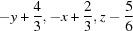
; (iii) 
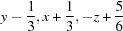
; (iv) 
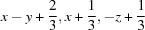
; (v) 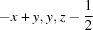; (vi) 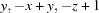.

**Table 2:** Dtails exprimentaux

Donnes crystallines	
Formule chimique	Na_9_Cr(MoO_4_)_6_
*M*_r_	1218,55
Systme cristallin, groupe d’espace	Trigonal, *R* 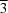 *c*
Temprature (K)	298
*a*, *c* ()	14,707(5), 19,175(7)
*V* (^3^)	3592(2)
*Z*	6
Type de rayonnement	Mo *K*
(mm^1^)	3,74
Taille des cristaux (mm)	0,22 0,16 0,10

Collection de donnes	
Diffractomtre	EnrafNonius CAD-4
Correction d’absorption	scan (North *et al.*, 1968 ▸)
*T*_min_, *T*_max_	0,481, 0,676
Nombre de rflexions mesures, indpendantes et observes [*I* > 2(*I*)]	2549, 875, 678
*R*_int_	0,065
(sin /)_max_ (^1^)	0,638

Affinement	
*R*[*F* ^2^ > 2(*F* ^2^)], *wR*(*F* ^2^), *S*	0,028, 0,078, 1,13
Nombre de rflexions	875
Nombre de paramtres	63
_max_, _min_ (e ^3^)	0,60, 0,57

Programmes informatiques: *CAD-4 EXPRESS* (Duisenberg, 1992 ▸; Macek Yordanov, 1992 ▸), *XCAD4* (Harms Wocadlo, 1995 ▸), *SHELXS97* et *SHELXL97* (Sheldrick, 2008 ▸), *DIAMOND* (Brandenburg Putz, 1999 ▸) et *WinGX* (Farrugia, 2012 ▸).

## supplementary crystallographic information

### Crystal data

**Table:**

Na_9_Cr(MoO_4_)_6_	*D*_x_ = 3.380 Mg m^−^^3^
*M**_r_* = 1218.55	Mo *K*α radiation, λ = 0.71073 Å
Trigonal, *R*3*c*	Cell parameters from 25 reflections
Hall symbol: -R 3 2"c	θ = 10–15°
*a* = 14.707 (5) Å	µ = 3.74 mm^−^^1^
*c* = 19.175 (7) Å	*T* = 298 K
*V* = 3592 (2) Å^3^	Prism, red
*Z* = 6	0.22 × 0.16 × 0.10 mm
*F*(000) = 3402	

### Data collection

**Table:**

Enraf–Nonius CAD-4 diffractometer	678 reflections with *I* > 2σ(*I*)
Radiation source: fine-focus sealed tube	*R*_int_ = 0.065
Graphite monochromator	θ_max_ = 27.0°, θ_min_ = 2.7°
ω/2θ scans	*h* = −16→4
Absorption correction: ψ scan (North *et al.*, 1968)	*k* = −17→1
*T*_min_ = 0.481, *T*_max_ = 0.676	*l* = −24→24
2549 measured reflections	2 standard reflections every 2 reflections
875 independent reflections	intensity decay: 1.2%

### Refinement

**Table:**

Refinement on *F*^2^	Primary atom site location: structure-invariant direct methods
Least-squares matrix: full	Secondary atom site location: difference Fourier map
*R*[*F*^2^ > 2σ(*F*^2^)] = 0.028	*w* = 1/[σ^2^(*F*_o_^2^) + (0.0286*P*)^2^ + 21.2743*P*] where *P* = (*F*_o_^2^ + 2*F*_c_^2^)/3
*wR*(*F*^2^) = 0.078	(Δ/σ)_max_ < 0.001
*S* = 1.13	Δρ_max_ = 0.60 e Å^−^^3^
875 reflections	Δρ_min_ = −0.57 e Å^−^^3^
63 parameters	Extinction correction: *SHELXL*, Fc^*^=kFc[1+0.001xFc^2^λ^3^/sin(2θ)]^-1/4^
0 restraints	Extinction coefficient: 0.00013 (4)

### Special details

**Table:**

Geometry. All e.s.d.'s (except the e.s.d. in the dihedral angle between two l.s. planes) are estimated using the full covariance matrix. The cell e.s.d.'s are taken into account individually in the estimation of e.s.d.'s in distances, angles and torsion angles; correlations between e.s.d.'s in cell parameters are only used when they are defined by crystal symmetry. An approximate (isotropic) treatment of cell e.s.d.'s is used for estimating e.s.d.'s involving l.s. planes.
Refinement. Refinement of *F*^2^ against ALL reflections. The weighted *R*-factor *wR* and goodness of fit *S* are based on *F*^2^, conventional *R*-factors *R* are based on *F*, with *F* set to zero for negative *F*^2^. The threshold expression of *F*^2^ > σ(*F*^2^) is used only for calculating *R*-factors(gt) *etc*. and is not relevant to the choice of reflections for refinement. *R*-factors based on *F*^2^ are statistically about twice as large as those based on *F*, and *R*- factors based on ALL data will be even larger.

### Fractional atomic coordinates and isotropic or equivalent isotropic displacement parameters (Å^2^)

**Table:**

	*x*	*y*	*z*	*U*_iso_*/*U*_eq_	
Mo1	0.51969 (4)	0.66053 (4)	0.02393 (2)	0.01759 (19)	
Cr1	0.3333	0.6667	0.9167	0.0096 (4)	
Na1	0.1089 (2)	0.6667	0.9167	0.0285 (8)	
Na2	0.5617 (2)	0.6706 (2)	0.21706 (11)	0.0334 (6)	
O1	0.2729 (3)	0.7346 (3)	0.85988 (19)	0.0177 (8)	
O2	0.4881 (4)	0.6129 (4)	0.1082 (2)	0.0342 (11)	
O3	0.0440 (4)	0.7277 (3)	0.8230 (2)	0.0267 (9)	
O4	0.0834 (3)	0.5458 (4)	0.8075 (2)	0.0312 (10)	

### Atomic displacement parameters (Å^2^)

**Table:**

	*U*^11^	*U*^22^	*U*^33^	*U*^12^	*U*^13^	*U*^23^
Mo1	0.0165 (3)	0.0218 (3)	0.0164 (3)	0.0110 (2)	−0.00230 (17)	0.00171 (18)
Cr1	0.0070 (5)	0.0070 (5)	0.0149 (8)	0.0035 (3)	0.000	0.000
Na1	0.0211 (11)	0.049 (2)	0.0250 (15)	0.0244 (12)	0.0073 (8)	0.0146 (16)
Na2	0.0341 (14)	0.0518 (18)	0.0200 (11)	0.0258 (14)	−0.0021 (10)	−0.0027 (11)
O1	0.0142 (18)	0.0155 (19)	0.0246 (18)	0.0083 (15)	−0.0003 (15)	0.0083 (15)
O2	0.037 (3)	0.041 (3)	0.0153 (18)	0.012 (2)	−0.0028 (18)	0.0009 (19)
O3	0.037 (3)	0.024 (2)	0.028 (2)	0.0221 (19)	−0.0003 (19)	−0.0009 (19)
O4	0.025 (2)	0.031 (2)	0.043 (2)	0.018 (2)	−0.0141 (19)	−0.014 (2)

### Geometric parameters (Å, º)

**Table:**

Mo1—O4^i^	1.723 (4)	Na1—O1	2.364 (4)
Mo1—O2	1.730 (4)	Na1—O3	2.407 (4)
Mo1—O3^ii^	1.745 (4)	Na1—O3^vii^	2.407 (4)
Mo1—O1^iii^	1.793 (4)	Na1—O4	2.649 (4)
Cr1—O1^iv^	1.966 (3)	Na1—O4^vii^	2.649 (4)
Cr1—O1	1.966 (3)	Na2—O2	2.308 (4)
Cr1—O1^v^	1.966 (3)	Na2—O2^ix^	2.384 (5)
Cr1—O1^vi^	1.966 (3)	Na2—O4^x^	2.414 (5)
Cr1—O1^vii^	1.966 (3)	Na2—O3^xi^	2.473 (5)
Cr1—O1^viii^	1.966 (3)	Na2—O3^x^	2.559 (4)
Na1—O1^vii^	2.364 (4)	Na2—O4^xi^	2.989 (5)
			
O4^i^—Mo1—O2	109.2 (2)	O3—Na1—O4^vii^	120.99 (15)
O4^i^—Mo1—O3^ii^	108.3 (2)	O3^vii^—Na1—O4^vii^	73.23 (14)
O2—Mo1—O3^ii^	108.2 (2)	O4—Na1—O4^vii^	157.6 (2)
O4^i^—Mo1—O1^iii^	111.80 (18)	O2—Na2—O2^ix^	98.5 (2)
O2—Mo1—O1^iii^	108.64 (19)	O2—Na2—O4^x^	112.11 (19)
O3^ii^—Mo1—O1^iii^	110.60 (18)	O2^ix^—Na2—O4^x^	110.62 (17)
O1^iv^—Cr1—O1	176.8 (2)	O2—Na2—O3^xi^	90.60 (17)
O1^iv^—Cr1—O1^v^	90.1 (2)	O2^ix^—Na2—O3^xi^	121.52 (18)
O1—Cr1—O1^v^	92.29 (15)	O4^x^—Na2—O3^xi^	118.75 (17)
O1^iv^—Cr1—O1^vi^	85.4 (2)	O2—Na2—O3^x^	164.06 (19)
O1—Cr1—O1^vi^	92.29 (15)	O2^ix^—Na2—O3^x^	92.00 (17)
O1^v^—Cr1—O1^vi^	92.29 (15)	O4^x^—Na2—O3^x^	74.78 (15)
O1^iv^—Cr1—O1^vii^	92.28 (15)	O3^xi^—Na2—O3^x^	73.64 (17)
O1—Cr1—O1^vii^	90.1 (2)	O2—Na2—O4^xi^	72.48 (16)
O1^v^—Cr1—O1^vii^	85.4 (2)	O2^ix^—Na2—O4^xi^	168.70 (19)
O1^vi^—Cr1—O1^vii^	176.8 (2)	O4^x^—Na2—O4^xi^	68.09 (17)
O1^iv^—Cr1—O1^viii^	92.28 (15)	O3^xi^—Na2—O4^xi^	66.40 (14)
O1—Cr1—O1^viii^	85.4 (2)	O3^x^—Na2—O4^xi^	98.30 (16)
O1^v^—Cr1—O1^viii^	176.8 (2)	Mo1—O2—Na2	135.6 (3)
O1^vi^—Cr1—O1^viii^	90.1 (2)	Mo1—O2—Na2^xii^	114.0 (2)
O1^vii^—Cr1—O1^viii^	92.28 (15)	Na2—O2—Na2^xii^	110.28 (17)
O1^vii^—Na1—O1	72.09 (19)	Mo1^xiii^—O3—Na1	108.60 (18)
O1^vii^—Na1—O3	158.89 (15)	Mo1^xiii^—O3—Na2^xiv^	111.7 (2)
O1—Na1—O3	90.55 (13)	Na1—O3—Na2^xiv^	101.42 (16)
O1^vii^—Na1—O3^vii^	90.55 (14)	Mo1^xiii^—O3—Na2^xv^	147.5 (2)
O1—Na1—O3^vii^	158.89 (15)	Na1—O3—Na2^xv^	100.80 (16)
O3—Na1—O3^vii^	108.7 (2)	Na2^xiv^—O3—Na2^xv^	74.53 (16)
O1^vii^—Na1—O4	89.66 (15)	Mo1^i^—O4—Na2^xv^	123.6 (2)
O1—Na1—O4	72.06 (14)	Mo1^i^—O4—Na1	117.1 (2)
O3—Na1—O4	73.23 (14)	Na2^xv^—O4—Na1	98.12 (17)
O3^vii^—Na1—O4	120.99 (15)	Mo1^i^—O4—Na2^xiv^	151.0 (2)
O1^vii^—Na1—O4^vii^	72.06 (14)	Na2^xv^—O4—Na2^xiv^	67.71 (15)
O1—Na1—O4^vii^	89.65 (15)	Na1—O4—Na2^xiv^	83.91 (14)

Symmetry codes: (i) −*x*+2/3, −*x*+*y*+1/3, −*z*+5/6; (ii) −*y*+4/3, −*x*+2/3, *z*−5/6; (iii) *y*−1/3, *x*+1/3, −*z*+5/6; (iv) *y*−1/3, *x*+1/3, −*z*+11/6; (v) −*y*+1, *x*−*y*+1, *z*; (vi) −*x*+*y*, −*x*+1, *z*; (vii) *x*−*y*+2/3, −*y*+4/3, −*z*+11/6; (viii) −*x*+2/3, −*x*+*y*+1/3, −*z*+11/6; (ix) *x*−*y*+2/3, *x*+1/3, −*z*+1/3; (x) −*x*+*y*, *y*, *z*−1/2; (xi) *y*, −*x*+*y*, −*z*+1; (xii) *y*−1/3, −*x*+*y*+1/3, −*z*+1/3; (xiii) −*y*+2/3, −*x*+4/3, *z*+5/6; (xiv) *x*−*y*, *x*, −*z*+1; (xv) −*x*+*y*, *y*, *z*+1/2.
